# Cerebral [^18^ F]T807/AV1451 retention pattern in clinically probable CTE resembles pathognomonic distribution of CTE tauopathy

**DOI:** 10.1038/tp.2016.175

**Published:** 2016-09-27

**Authors:** D L Dickstein, M Y Pullman, C Fernandez, J A Short, L Kostakoglu, K Knesaurek, L Soleimani, B D Jordan, W A Gordon, K Dams-O'Connor, B N Delman, E Wong, C Y Tang, S T DeKosky, J R Stone, R C Cantu, M Sano, P R Hof, S Gandy

**Affiliations:** 1 Fishberg Department of Neuroscience, Icahn School of Medicine at Mount Sinai, New York, NY, USA; 2 Friedman Brain Institute, Icahn School of Medicine at Mount Sinai, New York, NY, USA; 3 Department of Psychiatry, Icahn School of Medicine at Mount Sinai, New York, NY, USA; 4 Department of Nuclear Medicine, Icahn School of Medicine at Mount Sinai, New York, NY, USA; 5 Burke Rehabilitaiton Hospital, White Plains, NY, USA; 6 The NFL Neurological Program, Icahn School of Medicine at Mount Sinai, New York, NY, USA; 7 Department of Rehabilitation Medicine, Icahn School of Medicine at Mount Sinai, New York, NY, USA; 8 Department of Radiology, Icahn School of Medicine at Mount Sinai, New York, NY, USA; 9 Department of Neurology, University of Florida, Gainesville, FL, USA; 10 Department of Radiology and Medical Imaging, University of Virginia, Charlottesville, VA, USA; 11 Department of Neurosurgery, University of Virginia, Charlottesville, VA, USA; 12 Centre for the Study of Traumatic Encephalopathy, Boston University School of Medicine, Boston, MA, USA; 13 Department of Neurosurgery, Emerson Hospital, Concord, MA, USA; 14 Department of Neurology, Icahn School of Medicine at Mount Sinai, New York, NY, USA

## Abstract

Chronic traumatic encephalopathy (CTE) is a neurodegenerative disorder most commonly associated with repetitive traumatic brain injury (TBI) and characterized by the presence of neurofibrillary tangles of tau protein, known as a tauopathy. Currently, the diagnosis of CTE can only be definitively established postmortem. However, a new positron emission tomography (PET) ligand, [^18^F]T807/AV1451, may provide the antemortem detection of tau aggregates, and thus various tauopathies, including CTE. Our goal was to examine [^18^F]T807/AV1451 retention in athletes with neuropsychiatric symptoms associated with a history of multiple concussions. Here we report a 39-year-old retired National Football League player who suffered 22 concussions and manifested progressive neuropsychiatric symptoms. Emotional lability and irritability were the chief complaints. Serial neuropsychological exams revealed a decline in executive functioning, processing speed and fine motor skills. Naming was below average but other cognitive functions were preserved. Structural analysis of longitudinally acquired magenetic resonance imaging scans revealed cortical thinning in the left frontal and lateral temporal areas, as well as volume loss in the basal ganglia. PET with [^18^F]florbetapir was negative for amyloidosis. The [^18^F]T807/AV1451 PET showed multifocal areas of retention at the cortical gray matter–white matter junction, a distribution considered pathognomonic for CTE. [^18^F]T807/AV1451 standard uptake value (SUV) analysis showed increased uptake (SUVr⩾1.1) in bilateral cingulate, occipital, and orbitofrontal cortices, and several temporal areas. Although definitive identification of the neuropathological underpinnings basis for [^18^F]T807/AV1451 retention requires postmortem correlation, our data suggest that [^18^F]T807/AV1451 tauopathy imaging may be a promising tool to detect and diagnose CTE-related tauopathy in living subjects.

## Introduction

Traumatic brain injury (TBI) is associated with an increased risk of developing dementia, including Alzheimer's disease (AD).^1, 2^ However, recent research in postmortem samples suggests that a separate neurodegenerative syndrome, chronic traumatic encephalopathy (CTE), might be the cause of decline in some individuals who have sustained both single and multiple concussive and subconcussive blows to the head.^3, 4^ CTE, which was first observed in ‘punch drunk' boxers^5^ and termed ‘dementia pugilistica',^6^ is a neurodegenerative disease seen in individuals with a history of repetitive brain trauma, that is, blows to the head. CTE is characterized neuropathologically by progressive deposition of neurofibrillary tangles of hyperphosphorylated tau.^4^ Amyloidosis is a less consistent feature, present in only half of the cases of CTE examined at postmortem. The tauopathy shows a particular perivascular, sulcal, and neocortical distribution, distinguishing CTE from AD and other tauopathies.^4, 7, 8^ CTE also involves widespread axonal disruption, with eventual degeneration of the neocortex, hippocampus and other limbic structures, and basal forebrain.^7, 9^

Clinically, individuals with CTE can display symptoms that overlap with those seen in AD, such as impairments in memory and executive function, but, in contrast to AD, CTE is more frequently characterized by irritability, emotional lability, aggression, impulsivity, suicidality and substance abuse.^7, 10, 11^ Changes in mood and behavior typically precede cognitive decline in CTE, although clinical presentation is highly variable.^7, 11^Although individuals with CTE are at risk for severe functional impairment as well as disabling and life-threatening behavioral disturbances; currently, the disease can only be definitively diagnosed at postmortem.^11^ Because of this limitation, the discovery of reliable and valid body fluid and/or neuroimaging biomarkers is crucial to developing diagnostic specificity and identifying the underlying pathology in order to track and treat these individuals during life. Current estimates of prevalence based solely on neuropsychological exams or solely on neuropathology are each inherently susceptible to certain biases that could be mitigated with a reliable molecular biomarker.

Currently, little is known about the progression of CTE pathology *in vivo.* Attempts to detect CTE in living individuals have been challenging. Clinical symptoms are often based on the retrospective reports of family members after the loss of a loved one with suspected pathology, and common signs overlap with those seen in other neurodegenerative disorders.^7, 12^ Standard neuroimaging often reveals no gross lesions.^13^ However, neuropsychological testing can detect subtle changes in cognition, and recent studies of retired athletes with a history of concussions have revealed focal impairments in episodic memory,^14, 15, 16^ executive function,^14^ naming^15^ and semantic fluency.^16^ In addition, the identification of biomarkers, such as levels of serum and cerebrospinal fluid (CSF) tau^17, 18^ and CSF neurofilament proteins,^18^ have shown promise in detecting peripheral changes that may appear when CTE or other tauopathy is present. Although studies examining blood and CSF levels of tau and other neuronal injury markers after acute TBI have been mixed (for reviews see refs 19, 20), a recent study of professional hockey players showed that total serum level of tau increased after concussion and was correlated with clinical outcome.^17^

Structural imaging can also aid in the diagnosis of CTE. Studies using diffuse tensor imaging have shown that diffusion abnormalities, representing microstructural white matter damage, may be associated with repetitive TBI (reviewed in ref. 13). In addition, a handful of cross-sectional magnetic resonance imaging (MRI) studies showing ventricular enlargement and cortical thinning after mild TBI,^21^ including in childhood athletes,^22^ retired professional athletes^16^ and military veterans,^23, 24^ appear to support postmortem findings. However, we are unaware of any longitudinal studies that have examined structural changes in MRI in individuals with a history of repetitive mild TBI.

Molecular imaging is also a promising tool in detecting CTE pathology *in vivo.* [^11^C]Pittsburgh compound B (PiB) amyloid imaging has revealed increased ligand binding following acute TBI, which appears to be greatest immediately (hours) following the injury and gradually decreases over time.^25^ The residual amyloid present in some individuals 1-year post-TBI may (dependent on their age) reflect slowed clearance of acutely deposited amyloid, evolution to AD or evolution to CTE. In addition, *in vivo* detection of brain deposits of tau, the hallmark of CTE, is now possible through the use of novel positron emission tomography (PET) radiotracers. One ligand, [^18^F]T807 (also known as AV1451), has a high affinity and selectivity for tau over amyloid-β peptide (Aβ)^26^ and has demonstrated utility in revealing tauopathy in a variety of pathologies, including AD, frontotemporal dementia and CTE.^27, 28, 29^ We have previously reported the case of a 71-year-old retired National Football League (NFL) player with suspected CTE and a brain PET scan showing [^18^F]T807/AV1451 retention in the globus pallidus, putamen, substantia nigra and hippocampus. [^18^F]florbetapir PET imaging was negative, excluding the presence of the cerebral amyloidosis thus also excluding the diagnosis of AD.^27^ Another study that used [^18^F]T807/AV1451 imaging in a patient at risk for CTE revealed focal [^18^F]T807/AV1451 retention in the left anterior temporal lobe but no evidence of amyloidosis based on [^11^C]PiB PET.^30^ Additional studies are needed to determine whether positive [^18^F]T807/AV1451 imaging: (1) reliably reflects *in vivo* tauopathy with sufficient specificity for that proteinopathy; (2) reveals patterns distinct from other tauopathies; and (3) correlates with current or future clinical status.

In this study, we present the case of a 39-year-old retired NFL player with severe functional complaints and suspected CTE whose [^18^F]T807/AV1451 imaging results are strikingly similar to well-established postmortem patterns of tau deposition,^4^ suggesting that this novel ligand may be useful in diagnosing CTE. In particular, the presence of perivascular tauopathy at the depths of the cerebral cortical sulci has been determined to be pathognomonic for CTE by a recent National Institute on Neurological Diseases and Stroke (NINDS) neuropathology.^4^ We also present longitudinal neuropsychological and structural MRI data revealing patterns that may aid in understanding disease progression and further inform the development of diagnostic criteria. Despite the promise of this technique and the potential import of our ‘index subject', it is important to note that definitive radiological–pathological correlation will be required in order to establish that CTE tauopathy is indeed the basis for cerebral [^18^F]T807/AV1451 retention in subjects such as the retired athlete presented herein.

## Materials and methods

### Clinical and experimental structural brain imaging

The 2011 MRI was obtained on a 3-Tesla Siemens Verio MR system (Siemens Medical Solutions, Erlangen, Germany) at a Quest Imaging Center. Sequences included high-resolution T1-weighted MPRAGE images (TR=2300 ms, TE=3.1 ms, 160 sagittal slices, 1.2 mm slice thickness, voxel-size of 1.33 × 1.33 × 1.2 mm^3^, flip angle=9°). The 2015 MRI was obtained on a 3-Tesla Siemens Biograph MR system at the Icahn School of Medicine at Mount Sinai (ISMMS) Hess Center for Science and Medicine. Comparable high-resolution T1-weighted MPRAGE images were acquired (TR=2300 ms, TE=2.98 ms, 176 sagittal slices, 1.2 mm slice thickness, voxel-size of 1 × 1 × 1.2 mm^3^, flip angle=9°). The duration of time between the two scans was 3.9 years. Longitudinal analysis of cortical thickness and subcortical volume was performed using the FreeSurfer reconstruction pipeline (http://surfer.nmr.mgh.harvard.edu, version 5.3.0). Processing of individual time points included removal of non-brain tissue, automated Talairach transformation and intensity normalization, and detection of gray matter/white matter and gray matter/cerebrospinal fluid boundaries. Cortical thickness was calculated as the distance between these tissue boundaries (white matter and pial surfaces). The reconstructed surface models produced by the pipeline were inspected visually for accuracy and corrected manually using FreeSurfer's FreeView software (http://surfer.nmr.mgh.harvard.edu, version 5.3.0) as needed. Longitudinal processing included creating an unbiased within-subject base template from both time points and then using common information from the template to perform atlas registration, spherical surface map creation, and subcortical parcellation to extract reliable volume and thickness estimates.^31, 32^

### Clinical and experimental molecular brain imaging

[^18^F]Florbetapir imaging was conducted with a Siemens mCT 40-slice 4RPET/CT camera (Siemens Healthcare, Erlangen, Germany). The patient was injected with 370 MBq (10 mCi) of [^18^F]florbetapir. Image acquisition began ~50 min post injection and lasted for 20 min. Images were acquired in three dimensions, using a one-frame and one-bed position TOF Dynamic All PASS filter. Reconstruction was performed with a 400 matrix utilizing iterative reconstruction, with 24 subsets and 4 iterations. The *z* axis filter was standard, summed images 5.0 mm full width/half maximum Gaussian filter was used. The field of view was 50 cm in diameter, with 74 total slices. [^18^F]T807/AV1451 imaging was acquired and reconstructed using the same parameters as the [^18^F]florbetapir scan. After an appropriate washout period, the patient was injected with 370 MBq (10 mCi) of [^18^F]T807/AV1451. Image acquisition began ~80 min post injection and lasted for 20 min. A total of 74 axial slices of 3 mm thickness were displayed for visual interrogation by a nuclear medicine physician.

For [^18^F]florbetapir analysis, standard uptake value (SUV) ratios (SUVr) were calculated by dividing the mean uptake in an analysis region by the mean uptake in a reference region. Six predefined anatomically relevant cortical regions of interest (frontal, temporal, parietal, precuneus, anterior cingulate and posterior cingulate) were used as analysis regions with the whole cerebellum used as a reference region.^33^ Calculations were performed using MIM software (MIM Software Version 6.1, MIM Software, Cleveland, OH, USA), which has a semi-automatic program for [^18^F]florbetapir SUVr calculation using three standard PET templates: normal, mild cognitive impairment and AD.^34^

For [^18^F]T807/AV1451 analysis, PET and T1-MPRAGE images were brain extracted using the FSL Brain Extraction Tool.^35^ Next, the subject's PET and MR were coregistered followed by alignment to the MNI brain template using FSL FLIRT.^36^ An MNI-Brodmann area atlas was used to extract mean PET intensity values within each area. In addition, mean PET intensity values were derived from subcortical region-of-interests defined by the Harvard–Oxford subcortical structural atlas and the Talairach atlas. In accordance with the standard practice of tauopathy PET, regional SUVrs were calculated using the cerebellar cortex as a reference region.^27, 37^

## Results

### Clinical presentation

**Figure 1:**
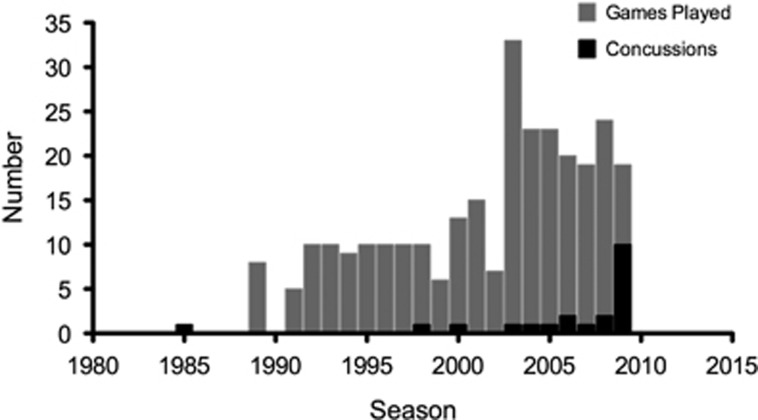
Total number of games played and recorded concussions by year. 1989–1994: high school football. 1995–1998: college football. 1999–2010: National Football League. The last concussion from the 2009 season was incurred in 2010.

A 39-year-old retired professional football player sustained 22 concussions, four of which resulted in loss of consciousness, during an 11-year career as an NFL player (Figure 1). He presented with complaints of irritability, emotional lability and cognitive decline. Following an informed consent process approved by the local institutional review board, he was evaluated at ISMMS in 2015 as part of a larger study comparing individuals with a history of multiple concussions to healthy controls and individuals with mild cognitive impairment with no concussion history. He also released to ISMMS investigators the medical records from his 2010 evaluation at the Boston University Center for Traumatic Encephalopathy. Evaluation at ISMMS included comprehensive neurologic and neuropsychological assessment, a structural 3 T MRI scan, a clinical [^18^F]florbetapir PET scan to determine the presence or absence of amyloid, and a research PET scan using the novel [^18^F]T807/AV1451 tau ligand to determine the possible presence of tauopathy.

Concussion history was determined based on an in-depth interview performed in 2010 by a neurologist specializing in TBI and CTE. An abbreviated version of the Brain Injury Screening Questionnaire^38^ was administered in 2015 and corroborated the 2010 report. Detailed information was obtained about reported concussions, including their approximate date, descriptions of the events, and the nature and duration of immediate post-concussion symptoms (for example, confusion, disorientation, retrograde and/or anterograde amnesia, loss of consciousness). Total number of games played was also recorded, as this information may reflect history of exposure to subconcussive impacts (Figure 1). Concussions were classified according to guidelines developed by the American Academy of Neurology.^39^On the basis of these criteria, the evaluators determined that the patient experienced 22 concussive events, 20 of which were Grade II, one Grade I, and one Grade III. Four of the concussive events resulted in loss of consciousness. The last recorded concussion occurred ~9 months before the 2010 neuropsychological testing. Detailed neurological examinations were conducted in 2010 and 2015 and were normal. In addition, *APOE* genotyping from 2010 showed a genotype of *ɛ3/ɛ3.*

Over the past several years, including prior to his retirement, the subject noticed difficulties with concentration and memory, particularly in short-term memory; the severity of his deficit was sufficient to affect his activities of daily living. In addition to memory complaints, the subject also reported numerous behavioral and functional symptoms such as increased agitation, irritability, impulsivity, migraine headaches, sleep disturbances, and increased sensitivity to noise. At the time of the 2010 evaluation, he reported experiencing ‘mild' feelings of being slowed cognition, headache irritability, poor concentration, and sensitivity to light. In the 2015 evaluation in clinical interview, the most prominent symptom was irritability. He also reported depression that occurred within the last 2 years and anxiety. The Beck Depression Inventory from 2015 was 24, indicating moderate depression. The Neuropsychiatric Inventory was completed in 2015 by his wife who endorsed severe irritability, moderate agitation/aggression, depression/dysphoria, disinhibition and sleep disturbance and mild delusional beliefs, anxiety and elation. His Clinical Dementia Rating (CDR) from 2015 was 0.5 and mild problems with memory, judgment and problem solving, community affairs and home and hobbies were endorsed. A functional questionnaire from 2015 identified difficulty with assembling tax and business records, shopping alone, remembering to turn off the stove and keeping appointments.

### Clinical and research neuropsychological assessment

Neuropsychological status was assessed in 2010 and 2015 and with structured surveys including: the Rivermead Post Concussion Symptom Questionnaire, the Beck Depression Inventory (BDI), the Neuropsychiatric Inventory (NPI), the CDR and the Functional Activities Questionnaire (FAQ).

The patient had 16 years of education. Premorbid intelligence, assessed by the Wide Range Achievement Test, was estimated to be in the superior range. Using available norms, neuropsychological tests, both from current assessment and previous testing identified in the medical records, were reported as *z*-scores. Using this method, comparison of scores from 2010 to 2015 showed a decline in executive functioning, processing speed and fine motor function (Table 1). Additional tests given only in 2015 showed below-average performance only in lexical retrieval. Performance was average or above average for long- and short-term verbal memory, working memory and semantic fluency, despite self-reported severe impairment with working memory and distractibility.

### Clinical and experimental structural brain imaging

Extended clinical and experimental structural brain imaging methods appear in the Materials and Methods section earlier in the text.

**Figure 2:**
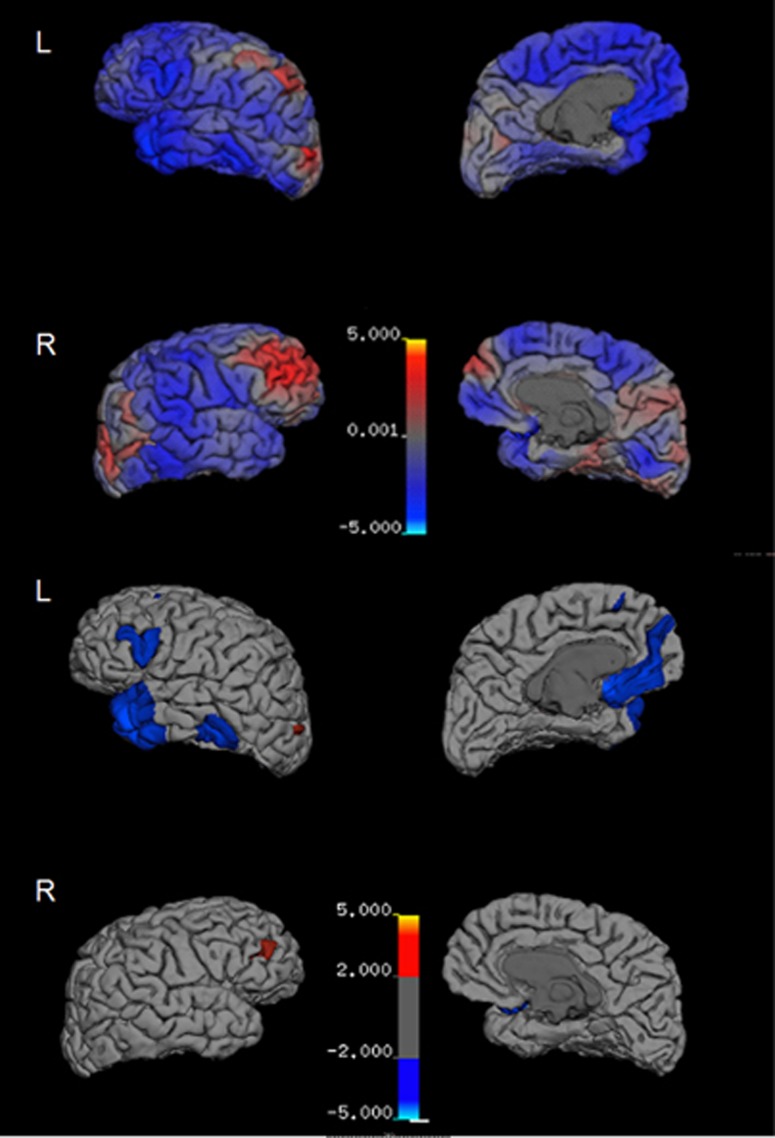
Percent change in cortical thickness from 2011 to 2015. Map of cortical thinning across the brain hemispheres are presented with dark gray regions representing sulci and light gray regions representing gyri. The top panel displays changes in thickness from 0 to 5% with no cutoff. The bottom panel displays changes in thickness >2%. Color scales represent percent thickness change relative to 2011 from 0 to ±5%.

**Figure 3:**
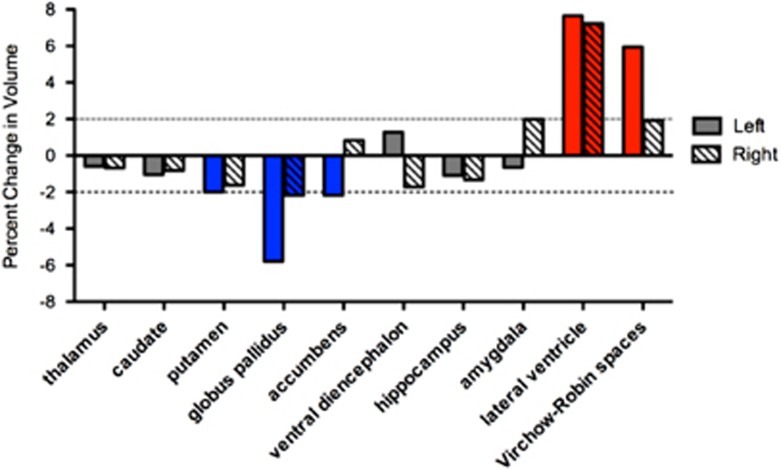
Percent change in subcortical volumes from 2011 to 2015.

Both the 2011 and the 2015 MRI were evaluated by a neuroradiologist and were read clinically as within normal limits with no obvious atrophy or lesions. Comparison cortical thickness measures derived from 3D T1 weighted MRI sequences acquired in 2011 and 2015 showed diffuse cortical thinning in both hemispheres (Figure 2), with the greatest thinning (>2% change) in the left inferior frontal gyrus corresponding to Broca's area, medial orbitofrontal cortex, mid-temporal gyrus, and the temporal pole (Figure 2). Thickness increases >2% were found in the left lateral occipital gyrus and the right rostral frontal gyrus (Figure 2). Comparison of 2011 and 2015 MRI scans also showed a negative trend with respect to volume of deep gray matter structures, with the largest decreases seen in the basal ganglia (globus pallidus, putamen and nucleus accumbens). Increases in volume were found in bilateral lateral ventricles and in summed left hemisphere Virchow–Robin spaces (Figure 3).

### Clinical and experimental molecular brain imaging

Extended clinical and experimental molecular brain imaging methods appear in the Materials and Methods section earlier in the text.

The clinical [^18^F]florbetapir scan was rated by nuclear medicine physicians trained to interpret [^18^F]florbetapir images using a binary (positive or negative) visual approach.^40^ Transaxial, coronal and sagittal images were examined. The scan results were considered positive if uptake in the cerebral gray matter equaled or exceeded the uptake in the white matter in at least two major areas of the brain. A positive [^18^F]florbetapir scan indicates moderate-to-frequent fibrillar amyloid plaques; a negative [^18^F]florbetapir scan indicates sparse-to-no fibrillar amyloid plaques, which is inconsistent with a diagnosis of AD. In our subject the [^18^F]florbetapir PET scan was negative for cerebral amyloidosis (Figure 3), thereby excluding AD as a cause of his cognitive decline. The global cortical SUVr calculated for the [^18^F]florbetapir scan was 0.929, which is below the published cutoff of 1.1 and reflects absence of amyloidosis.^41, 42, 43^

**Figure 4:**
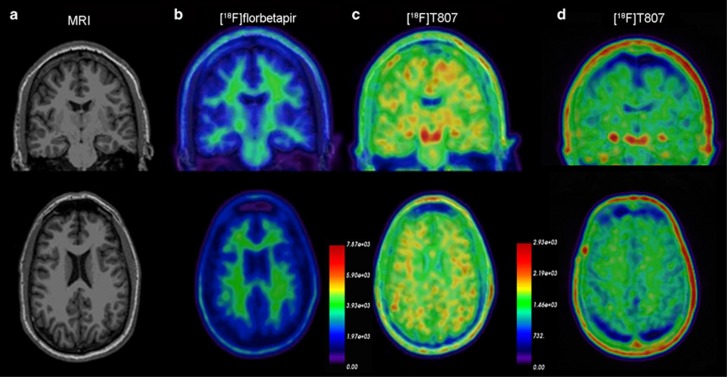
PET Imaging from a 39-year-old retired NFL player. (**a**) structural MRI image, (**b**) [^18^F]florbetapir PET (**c**) [^18^F]T807/AV1451 PET, (**d**) [^18^F]T807/AV1451 PET from a healthy age-matched control subject. Note that the [^18^F]florbetapir image was negative for amyloid accumulation, while the [^18^F]T807/AV1451 image shows extensive cortical ligand retention, especially at the junction of the gray and white matter, as is characteristic of the distribution of tauopathy in CTE. The PET scales represent ligand uptake in Bq/ml. CTE, chronic traumatic encephalopathy; MRI, magnetic resonance imaging; PET, positron emission tomography.

Experimental tauopathy imaging was performed using [^18^F]T807/AV1451. As noted above, this ligand has a high affinity and selectivity for tauopathy over fibrillar deposits of Aβ^26^ and has demonstrated utility in revealing tauopathy in a variety of pathologies, including AD, frontotemporal dementia and CTE.^27, 28, 29^ Visual assessment of the [^18^F]T807/AV1451 PET scan revealed multiple areas of retention of [^18^F]T807/AV1451 throughout the cerebral cortex particularly at the gray–white matter junctions (Figure 4). Signal increases were also apparent in the midbrain, globus pallidus, and hippocampus. In addition, quantification of the scan was performed by spatially normalizing the PET image to a T1 MRI structural image and applying region-of-interest templates (see Materials and Methods) for extraction on regional SUVs. Brain SUVs were divided by the cerebellar cortex SUV to calculate SUVr in multiple cortical and subcortical regions (Table 2). Because no standard SUVr cutoff value has been established for [^18^F]T807/AV1451 imaging in CTE or other conditions, we used the cutoff of 1.1 established in the AD [^18^F]florbetapir literature^41, 42, 43^ to identify regions of increased ligand uptake. This cutoff is likely conservative for [^18^F]T807/AV1451, as cerebellar deposition of tau in CTE^7^ will lead to underestimated SUV ratios; indeed, in the current individual, [F^18^]T807/AV1451 ligand uptake was seen in the cerebellum. Cortical regions of increased uptake were found in cingulate cortex (Brodmann areas 23, 24), retrosplenial cortex (areas 26, 29, 30), occipital cortex (areas 17, 18, 19), primary auditory (areas 41, 42), parietotemporal gyrus (area 39)^44^ the temporal pole (area 38), hippocampal formation (areas 27, 34) and the lateral orbitofrontal cortex (area 47); these localized findings were similar bilaterally. Subcortical regions of increased uptake were found in the putamen, globus pallidus, hippocampus, nucleus accumbens, and substantia nigra, again with similar findings in both hemispheres. Several of these regions (temporal pole, orbitofrontal cortex, putamen, globus pallidus and nucleus accumbens), corresponded to the areas of cortical thinning and subcortical volume loss observed in the structural analyses (Figures 2 and 3). Other investigators using several tau ligands including [F^18^]T807/AV1451 have observed similar retention patterns, and they, too, have interpreted this retention as nonspecific and/or due to local concentrations of tissue monamine oxidase.^44, 45^ Indeed, in a typical healthy control studied at our site, nonspecific ligand uptake was observed in the substantia nigra and hippocampus, with no cortical uptake.

## Discussion

We present the case of a 39-year-old retired professional athlete with a history of multiple concussions that developed emotional lability, behavioral disturbances and cognitive impairment. Although neuropsychological testing revealed high levels of function in multiple domains, executive function, processing speed, and motor speed declined over that time, and naming was below average. This pattern is similar to findings from previous neuropsychological studies in retired athletes with a history of concussions.^14, 15, 16^ The patient performed in the average-to-superior range on several tests of short-term memory, despite reporting problems with working memory in his daily life. Difficulties with attention and distractibility may explain these complaints, as he reported attention and concentration problems and was medically treated for these symptoms. Further neuropsychological testing specifically examining aspects of attention may disambiguate the underlying cause of poor performance. The neuropsychological battery identified a pattern different from other neurodegenerative diseases such as AD (unlikely at age 39 years). The presence of aggression, irritability and disinhibition reflect a triad of seriously disabling symptoms. In addition, relatively new onset of depression with anxiety was noted. Altogether, these neuropsychological and psychiatric symptoms undoubtedly contribute to the significant functional impairment reported in 2015.

Analysis of structural brain changes suggests diffuse atrophy, most prominently seen within the frontal lobe, basal ganglia and lateral temporal lobe, with apparent sparing of the medial temporal lobes. Thinning in Broca's area (Figure 2), which has been implicated in word production and lexical retrieval,^46, 47^ may account for the striking impairment seen in the Boston Naming Test, one of the patient's lowest scores. Circuits involving the frontal cortex and the basal ganglia have also been shown to be involved in lexical retrieval,^48^ as well as executive function.^49, 50^ As such, the atrophy seen in the basal ganglia and the medial and lateral frontal cortex may contribute to the patient's difficulties in these domains and may contribute to the reduced motor speed and feeling ‘slowed down' along with other disabling behavioral symptoms. Thinning in the left orbitofrontal cortex and temporal pole (Figure 2) may underlie the patient's complaint of impulsivity. A recent study examining athletes with a history of multiple concussions found that cortical thinning in the orbitofrontal cortex and temporal pole (and decreased diffusivity in the uncinate fasciculus, the white matter tract that connects them) correlated with errors on a go/no-go task and a measure of aggression.^51^ Little hippocampal or medial temporal lobe atrophy was found, consistent with the high scores seen in the delayed recall tests. Overall, these structural findings are in accord with the few brain-wide cross-sectional studies wherein cortical thickness was examined in brain images from subjects exposed to sports or military-related mild TBI.

The pattern of ligand binding in the [^18^F]T807/AV1451 tau PET scan suggests extensive tauopathy deposition, particularly at the gray–white matter boundary. We were especially struck by the apparent similarity of [^18^F]T807/AV1451 retention in this subject with the recent recognition by an NINDS CTE neuropathology consensus panel that tauopathy at the depths of the cerebral cortical sulci is pathognomonic for CTE.^4^ This single feature of our PET images is important not only for its apparent mirroring of the hallmark distribution of the tauopathy of CTE, but this juxtacortical distribution of ligand retention does not obviously invoke an immediate concern regarding off-target ligand retention.^52^ Indeed, although the signal in the globus pallidus may reflect nonspecific retention of [^18^F]T807/AV1451,^52^ the accompanying volume decreases seen in the globus pallidus of this patient suggest involvement of this structure in his disease process. These areas may contribute to the behavioral dysregulation noted in this patient. Our previous report of a 71-year-old retired NFL player who also had a negative amyloid scan did exhibit more focal [^18^F]T807/AV1451 binding in basal ganglia and hippocampus with minimal binding in the neocortex.^27^ That patient presented with hypomimia, greater memory impairment and fewer behavioral symptoms than the present case. The findings in the present subject with relatively mild to moderate symptoms suggest that this ligand may be useful in detecting CTE tauopathy *in vivo*, in at least some patients. The success in visualizing [^18^F]T807/AV1451 retention in a pattern that is associated with a singular neuropathological signature in this 39-year-old subject with mild to moderate symptoms is especially important in light of recent pathologically confirmed case of CTE in a 25-year-old former college football player.^3, 5, 53^ Both the current case and the 25-year-old case published by Mez *et al.* showed contemporaneous clinical signs of CTE. Our patient also emphasizes the prominent psychiatric component of the presentation and the recovery of a striking image despite highly functional status. This raises the possibility that tauopathy imaging could inform decisions of whether to initiate or continue engagement in high-risk activities and/or or as an outcome for anti-tauopathy therapies to interrupt the degenerative process. We would like to restate the point that despite the promise of this technique and the potential import of our ‘index subject', it is important to note that definitive radiological–pathological correlation will be required in order to establish that CTE tauopathy is indeed the basis for cerebral [^18^F]T807/AV1451 retention in subjects such as the retired athlete presented herein. The achievement of this goal with [^18^F]T807/AV1451 is more challenging than was the case with florbetapir in AD. CTE patients are often younger than AD patients, and encountering CTE patients within the final year of life so that one might obtain both [^18^F]T807/AV1451 imaging and postmortem neuropathology from the same subject will not be easily achieved.

Another recent study using a different ligand, [^18^F]FDDNP, reported that professional athletes with a history of multiple concussions had increased ligand retention compared with controls in several cortical and subcortical regions, including the medial temporal lobe, striatum and frontal cortex. Only increased signal in the midbrain and amygdala were found in all 14 of the athletes.^37^ All athletes but one in this study were also diagnosed with mild cognitive impairment, but patterns of [^18^F]FDDNP uptake were able to distinguish between the athletes and an AD group, particularly in subcortical regions, whereas cortical regions, particularly the medial temporal lobe, showed overlap between the CTE and AD groups. However, participants in this study did not undergo amyloid PET scans, an important point because [^18^F]FDDNP is reported to have a relatively low affinity for tauopathy neurofibrillary tangles and a low specificity for tauopathy over amyloid;^54, 55, 56^ indeed, [^18^F]FDDNP is considered to bind to some degree to both amyloidosis and tauopathy. Thus, further research using agents like [^18^F]T807/AV1451 and [^18^F]florbetapir that have a high selectivity and specificity for particular proteinopathies are needed to clarify the burden of tauopathy and amyloidosis separately, both to understand the disease process better and to facilitate differential diagnosis. Indeed, one recent study that examined [^18^F]T807/AV1451 in an MCI/AD cohort revealed a pattern of tauopathy deposition that was concentrated mainly in the inferior temporal gyrus and corresponded to Braak staging regional distribution,^57^ distinct from the pattern found here, suggesting that [^18^F]T807/AV1451 may be useful not only in detecting tauopathy but in differentiating among tauopathies. These are early days in the *in vivo* detection of these complex degenerative disorders, and much additional investigation is required. To that end, though we have primarily discussed [^18^F]T807/AV1451, however, there are a host of other tauopathy ligands in the pipeline. For a review on Tau PET imaging see refs 58, 59.

In closing, we would like to restate the point that, despite the promise of this technique and the potential import of our ‘index subject', it is important to note that definitive radiological–pathological correlation will be required in order to establish that CTE tauopathy is indeed the basis for cerebral [^18^F]T807/AV1451 retention in subjects such as the retired athlete presented herein. As also noted above, the achievement of this goal with [^18^F]T807/AV1451 is more challenging than was the case with florbetapir in AD. CTE patients are often younger than AD patients, and encountering CTE patients within the final year of life so that one might obtain both [^18^F]T807/AV1451 imaging and postmortem neuropathology from the same subject will not be easily achieved.

This case provides a window into the neuropsychiatric and structural progression of CTE and suggests that [^18^F]T807/AV1451 tauopathy imaging is a potentially promising tool to detect CTE-related neuropathology *in vivo* in at least some patients. Further research comparing [^18^F]T807/AV1451 results in CTE to healthy controls and other tauopathies will clarify whether there is a signature pattern that can be attributed to CTE, and thus whether and under what conditions tauopathy imaging can be used clinically to identify and track progression of CTE-related neurodegeneration.

## Figures and Tables

**Table 1:** Neuropsychological scores and percentiles

	*2010*			*2015*		
	*Score*	*Percentile*	Z-*score*	*Score*	*Percentile*	Z-*score*
*General intelligence*						
WAIS-IV	128^a^	97	1.88			
WRAT-IV				123^a^	97	1.88
						
*Verbal reasoning*						
WAIS-IV similarities	13^a^	84	1	14^a^	91	1.33
						
*Immediate and working memory*						
BVMT trial total	31	82	0.92	35	97	1.88
CVLT-II trial total				68	97	1.88
WAIS-IV digit span forward				11	50	0
						
*Delayed memory*						
BVMT delayed recall	11	73	0.061	12	90	1.28
CVLT-II LDFR				16	93	1.48
						
*Language*						
COWA FAS	57	77	0.074	50	65	0.39
Animals				32	90	1.28
BNT				55	19	-0.88
						
*Motor function*						
GPB dominant	53	84	1	64.5	34	**−0.41**^b^
GPB non-dominant	69	25	**−**0.67	90.6	2	**−2.05**^b^
						
*Executive function*						
Trail making test - B	24	100	3	46	78	**0.77**^b^
Stroop color-word	WNL			57	81	0.88
WAIS-IV digit span backward				11	70	0.66
WAIS-IV letter-number sequencing				20	50	0
						
*Processing speed*						
WAIS-IV digit symbol/coding	13^a^	84	1	11^a^	63	0.33
WAIS-IV symbol search	18^a^	100	2.66	14^a^	91	**1.33**^b^

Abbreviations: BNT, Boston Naming Test; BVMT, Brief Visuo-Spatial Memory Test; CVLT-II LDFR, California Verbal Learning Test II Long Delay Free Recall; COWA, Controlled Oral Word Association; GPB, Grooved Pegboard; WAIS-IV, Wechsler Adult Intelligence Scale, 4th edition; WNL, within normal limits; WRAT-IV, Wide Range Acheivement Test 4.

a Scaled score, all other scores reported as raw scores.

b *Z*-score change of ⩾1. Scores were not available for all tests for both time points.

**Table 2:** SUV ratios for [^18^F]T807 scan

*Atlas*	*Region*	*SUV ratio*	
		*Left*	*Right*
MNI-Brodmann Atlas	18	1.122	1.139
	19	1.112	1.150
	23	1.191	1.195
	24	1.186	1.139
	26	1.209	1.163
	27	1.168	1.137
	29	1.145	1.156
	30	1.110	1.027
	32	1.152	1.187
	34	1.130	1.097
	37	1.164	1.135
	41	1.209	1.170
	47	1.154	1.168
Harvard–Oxford Subcortical Atlas	Thalamus	1.162	1.131
	Caudate nucleus	1.064	1.025
	Putamen	1.229	1.199
	Globus pallidus	1.331	1.299
	Hippocampus	1.185	1.169
	Amygdala	1.071	1.076
	Nucleus accumbens	1.107	1.167
Talaraich Atlas	Pons	1.080	1.041
	Midbrain (substantia nigra)	1.375	1.364

Abbreviation: SUV, standard uptake value.

Note, for Brodmann area analysis we are only showing regions with ratios above the cutoff of 1.1. All Brodmann areas were analyzed.
